# *Past1* Modulates *Drosophila* Eye Development

**DOI:** 10.1371/journal.pone.0169639

**Published:** 2017-01-06

**Authors:** Orly Dorot, Hermann Steller, Daniel Segal, Mia Horowitz

**Affiliations:** 1 Department of Cell Research and Immunology, George S. Wise Faculty of Life Sciences, Tel Aviv University, Ramat Aviv, Israel; 2 Howard Hughes Medical Institute, Strang Laboratory of Cancer Research, The Rockefeller University, New York, New York, United States of America; 3 Department of Molecular Microbiology and Biotechnology and the Interdisciplinary Sagol School of Neurosciences, George S. Wise Faculty of Life Sciences, Tel Aviv University, Ramat Aviv, Israel; University of Dayton, UNITED STATES

## Abstract

Endocytosis is a multi-step process involving a large number of proteins, both general factors, such as clathrin and adaptor protein complexes, and unique proteins, which modulate specialized endocytic processes, like the EHD proteins. EHDs are a family of Eps15 Homology Domain containing proteins that consists of four mammalian homologs, one *C*. *elegans*, one *Drosophila melanogaster* and two plants orthologs. These membrane-associated proteins are involved in different steps of endocytic trafficking pathways. We have previously shown that the *Drosophila* EHD ortholog, PAST1, associates predominantly with the plasma membrane. Mutations in *Past1* result in defects in endocytosis, male sterility, temperature sensitivity and premature death of the flies. Also, *Past1* genetically interacts with *Notch*. In the present study, we investigated the role of PAST1 in the developing fly eye. In mutant flies lacking PAST1, abnormal differentiation of photoreceptors R1, R6 and R7 was evident, with partial penetrance. Likewise, five cone cells were present instead of four. Expression of transgenic PAST1 resulted in a dominant negative effect, with a phenotype similar to that of the deletion mutant, and appearance of additional inter-ommatidial pigment cells. Our results strongly suggest a role for PAST1 in differentiation of photoreceptors R1/R6/R7 and cone cells of the fly ommatidia.

## Introduction

*Drosophila* PAST1 is an endocytic protein, which belongs to the family of Eps15 homology (EH) domain-containing proteins. The *Past1* gene encodes two transcripts, RNA-A and RNA-B. The corresponding proteins, PAST1A and PAST1B, differ in seven amino acids missing from the N-terminus of PAST1B [1]. The two *Past1* transcripts are differentially expressed both temporally and spatially during the life cycle of the fly [2]. *Past1*B is expressed throughout development in both males and females, while *Past1*A is only expressed from the third larval stage onwards, and is restricted to the male testes [2]. *Past1* null mutant flies, generated by imprecise excision of a P-element, reach adulthood, but are temperature sensitive, infertile and die precociously. As expected, these flies present a defect in endocytosis. *Past1* shows genetic interactions with *Notch* pathway genes [2]. Recently it has been shown that PAST1 controls postsynaptic membrane elaboration and synaptic function [3].

**Fig 1:**
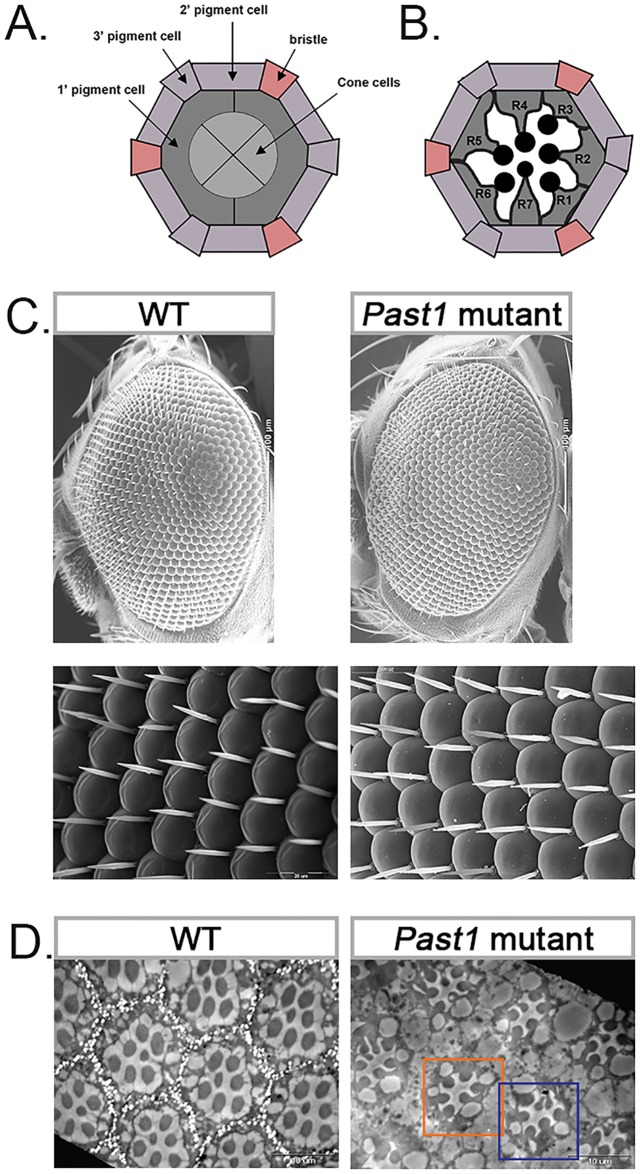
*Past1* mutant flies contain an abnormal number of photoreceptors in their eyes. (A) Schematic representation of the apical section of an ommatidium. (B) Schematic representation of a cross-section of an ommatidium. (C) Scanning electron microscopy images of eyes of wild type or homozygous null *Past1* mutant (*Past1*^*110-1*^) three-days-old adult flies. Upper panels x240, lower panels x1500. (D) Transmission electron microscopy images of eyes of wild type or *Past1*^*110-1*^ three-days-old adult flies. Orange frame delineates an example of an ommatidium with fewer photoreceptors than in the wild type. Blue frame delineates an example of an ommatidium with two potential R7 photoreceptors.

Endocytosis is a process responsible for downregulating signaling pathways, several of which control development of the fly eye [4, 5]. We, therefore, decided to search for a role of PAST1 in the developing fly eye. In the third instar larval eye disc, the first ommatidial cell to differentiate is photoreceptor R8. This photoreceptor promotes the differentiation of the surrounding undifferentiated cells into the seven additional photoreceptors (R1-R7) by secreting the ligand Spitz (Spi) (*Drosophila* EGF), which binds to DER (*Drosophila* EGFR) [6–8]. Upon completion of the differentiation of R8, R2/R5 and R3/R4 photoreceptors, a 90° rotation of the photoreceptor clusters takes place, to produce a mirror-image symmetry of ommatidia across the equator [9–14]. This process is followed by the formation of a second cluster of photoreceptors, comprised of R1/R6 and R7. Expression of the Delta ligand by differentiated R1/R6 induces the differentiation of R7, which in turn expresses Notch [15–19]. In addition, R7 expresses the receptors DER and Sevenless (Sev). The latter is activated by Bride of Sevenless (Boss), presented on the adjacent R8 cell [20–24]. Following specification of R7 photoreceptor, the four non-neuronal cone cells differentiate [13, 17, 19]. At early-mid pupal stage, each ommatidium is separated by the inter-ommatidial pigment cells (IPC) that undergo PCD thereby leaving six secondary and three tertiary pigment cells, a process mediated by Notch signaling [25–29]. Finally, at the mid pupal stage, the photoreceptors start to project their rhabdomeres [12, 30, 31] (Fig 1A and 1B).

Our results strongly suggest a role for PAST1 in differentiation of the ommatidia such that its absence or overexpression leads to abnormal differentiation of R1/R6/R7 and an aberrant number of both cone and pigment cells.

## Materials and Methods

### Fly strains

All strains were maintained and crosses were made on cornmeal molasses medium at 25°C. Fly stocks used were as follows: Wild type flies (Oregon-R), w [1118] and UAS-GFP, which served as a control. *Past1*^*110-1*^ is a null mutant of *Past1* (mutants were described in [2]. UAS-GFP-PAST1A and UAS-GFP-PAST1B transgenic flies were generated by standard embryo injections (BestGene Inc. CA, USA). GMRGal4, DaGal4 and mirrGal4 were obtained from Bloomington Drosophila Stock Center (Indiana University, IN, USA).

### Antibodies

The primary antibodies used in this study were: rabbit anti-PAST1 (anti-PAST1 antibodies as described in [2], mouse anti-actin (Sigma-Aldrich, Israel), and antibodies from the Developmental Studies Hybridoma Bank (University of Iowa, Iowa City, IA, USA) including rat anti-Elav (7E8A10, 1:100), mouse anti-Elav (9F8A9, 1:75), anti-Notch intracellular domain (1:25), mouse anti-Discs large (4F3, 1:50), mouse anti-Prospero (Prospero (MR1A), 1:50), mouse anti-cut (2B10, 1:100), mouse anti-Chaoptin (24B10, 1:100), and mouse anti-Rh1 (4C5, 1:50). Mouse anti-Boss (1:600) was a kind gift from Dr. H. Kramer, guinea pig anti-senseless (1:1000) was a kind gift from Dr. Hugo J. Bellen, rat anti-BarH1 (1:200) was a kind gift from Dr. Tiffany Cook. The secondary antibodies used in this study were Alexa488 conjugated secondary antibodies (Molecular Probes, Eugene, OR, USA), Cy2, Cy5 and horseradish peroxidase-conjugated goat anti-mouse and goat anti-rabbit (Jackson Labs, PA, USA).

### Immunofluorescence

Third instar larval eye disc or pupal and adult retinas were fixed in 4% paraformaldehyde for 20 and 60 minutes, respectively, followed by rinsing with PBT (1x PBS supplemented with 0.3% Triton X-100). First antibodies were diluted in BBT (1x PBS supplemented with 0.1% BSA, 0.1% Tween-20 and 250 mM NaCl) and were added for an overnight incubation at 4°C with shaking. Following several washes with PBT, secondary antibodies were added and incubated with shaking for 2 hours at room temperature (RT). After several washes with PBT the preparations were mounted with Galvanol. Cells were visualized using LSM510 Meta (ZEISS) confocal microscope.

For analysis of adult or pupal eye sections a minimum of 50 individual ommatidia were scored for each genotype. When using the GMRGal4-driver, all adult, pupae and larvae used were males, to avoid background effect in females homozygous for the GMRGal4.

### Endocytosis in Garland cells

Garland cells were dissected from third instar larvae and stained as previously described [2] Cells were visualized using LSM510 Meta confocal microscope.

Measurements of fluorescence intensity in individual cells were performed using the ImageJ software (http://rsb.info.nih.gov/ij/index.html).

### Electron Microscopy

For scanning electron microscopy (SEM), three-days-old adult flies were decapitated and their heads were fixed in 2.5% glutaraldehyde in PBS. They were then washed, dehydrated in graded ethanol solutions, dried from CO_2_ at critical point, coated with gold (Polaron SEM coating unit E5100) and examined with a Jeol JSM 840A SEM. Images were captured using scandium SEM imaging platform (Olympus).

For transmission electron microscopy (TEM), three-days-old adult flies were fixed in 2.5% glutaraldehyde in PBS. They were then washed, fixed in 1% OsO_4_ in PBS, and re-washed. After dehydration in graded ethanol solutions, the samples were embedded in Glycid ether (Serva, Heidelberg, Germany). Ultrathin sections were stained with uranyl acetate and lead citrate and examined with a Jeol 1200 EX TEM. Images were captured using SIS Megaview III and digitalized with the iTEM program the TEM imaging platform (Olympus).

### SDS/PAGE and western blotting

Flies (usually 10 flies in each preparation) were homogenized in RIPA lysis buffer (50 mM Tris-HCl, 150 mM NaCl, 1 mM EDTA, 1% Triton X-100, 1% sodium deoxycholate, 0.1% SDS), containing protease inhibitors (10 μg/ml leupeptin, 10 μg/ml aprotinin and 0.1 mM phenylmethylsulfonylfluoride, all from Sigma-Aldrich, Israel). Proteins were separated by 10% SDS-PAGE and transferred onto a nitrocellulose membrane (Schleicher and Schuell BioScience, Keene, NH, USA). After blocking in 5% skim milk and 0.1% Tween-20 in Tris-buffered saline (TBS) for 1 hour at RT, the membranes were incubated with the primary antibody for 1 hour at RT. The membranes were then washed three times in 0.1% Tween-20 in TBS and incubated with the appropriate secondary antibody for 1 hour at RT. After additional washes, membranes were reacted with ECL detection reagents (Santa Cruz Biotechnology, CA, USA) and analyzed by luminescent image analyzer (Kodak, Medical x-ray Processor 102, Rochester, NY, USA). The intensity of each band was measured by the GelQuant software (BiochemLabSolutions, San Francisco, CA, USA).

### Data analysis and quantification

Non-parametric, Mann-Whitney Test, was used for statistical analyses, unless otherwise indicated.

## Results

### The effect of null *Past1* mutation on fly eye development

**Fig 2:**
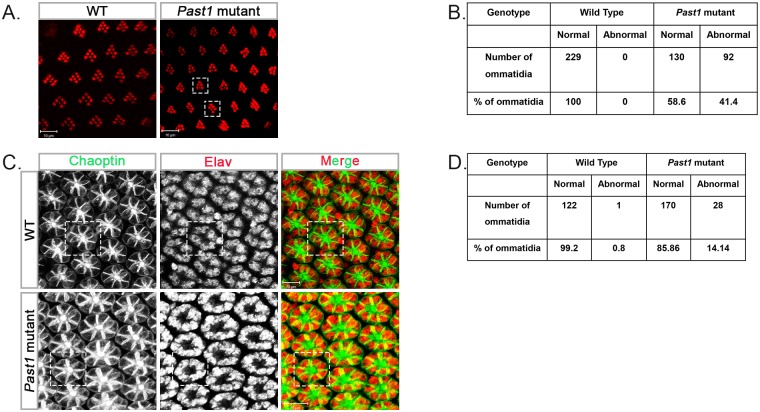
Ommatidial defects in *Past1* mutant flies. (A) Phalloidin staining of wild type or *Past1*^*110-1*^ homozygous mutant late pupa eyes. Dotted squares mark ommatidia with less than seven rhabdomeres. (B) Quantification of abnormal ommatidia (with less than seven rhabdomeres) in late pupal eyes of wild type (n = 229) and of *Past1*^*110-1*^ mutants (n = 222) ommatidia; *P* < 0.001. (C) Elav (red) and chapotin (green) staining of wild type and *Past1*^*110-1*^mutant early-mid pupa eyes (42-48h after puparium formation). The dotted square outlines an ommatidium with eight normal photoreceptors in wild type and an ommatidium with abnormal seven photoreceptors in *Past1*^*110-1*^mutant eyes. (D) Quantification of abnormal number of photoreceptors in early-mid pupa eyes of wild type (n = 198) and of *Past1*^*110-1*^ mutant (n = 123) ommatidia; *P* < 0.001.

To examine the possibility that PAST1 plays a role during eye development in *Drosophila*, we explored the effect of a null *Past1* mutation. No external morphological defects were detected by scanning electron microscopy in the eyes of three-days-old adults homozygous for a null *Past1* mutation compared to wild type flies (Fig 1C). Transmission electron microscopy analysis of *Past1* mutant eyes revealed some ommatidia with a decreased number of rhabdomeres, compared to wild type control (Fig 1D). In some of these abnormal ommatidia two potential R7 photoreceptors were observed (Fig 1D). To quantify this abnormality, staining of all the rhabdomers in late pupa eyes of both wild type and *Past1* mutants was performed using phalloidin. The results showed that 41% of the ommatidia in the *Past1* mutant eyes were abnormal, containing only six or five rhabdomeres (Fig 2A and 2B). We could confirm the abnormality in the number of photoreceptors in the developing *Past1* mutant eye at early-mid pupal stage, by staining with anti-chaoptin antibody, which at this stage is localized to the plasma membrane of all photoreceptors [32, 33]. The results (Fig 2C and 2D) indicated that 14% of the ommatidia at the early-mid pupal stage presented abnormal number of photoreceptors compared to the wild type.

**Fig 3:**
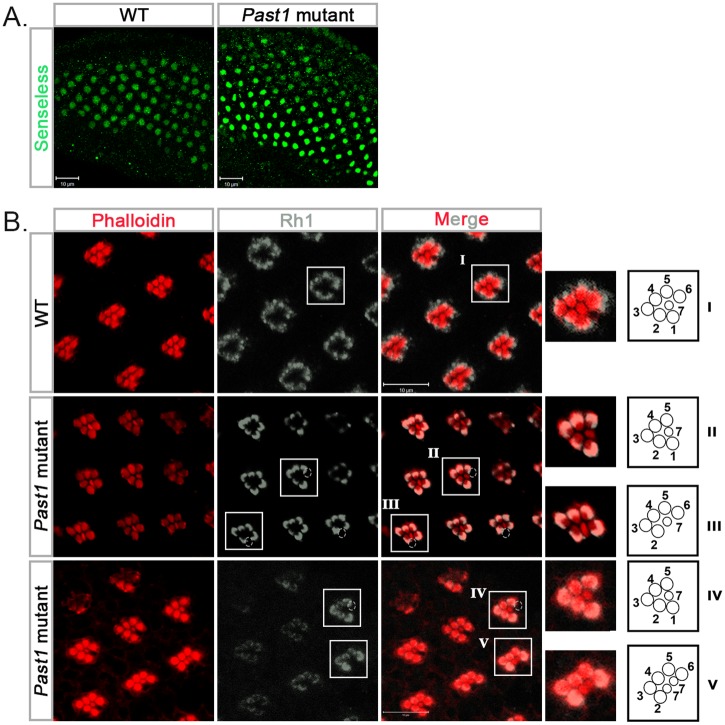
Photoreceptor differentiation in Past1 mutant fly eye. (A) Senseless staining of wild type and *Past1*^*110-1*^ homozygous mutant eye discs from third instar larvae. Shown are a Z-projections of confocal sections. (B) Phalloidin (red) and rhodopsin 1 (Rh1) (grey) staining of wild type *Past1*^*110-1*^ homozygous mutant late pupal eyes (85-96h after puparium formation). A schematic illustration depicting the localization of different photoreceptors in each zoomed-in image is shown on the right of each panel: I—ommatidium with normal seven photoreceptors (R1-R7), II—photoreceptor R6 is missing, III—photoreceptor R1 is missing, IV—photoreceptor R6 is missing, V—photoreceptor R1 is missing and there are two photoreceptors R7. The dotted circles outline the missing R1/R6 photoreceptors.

**Fig 4:**
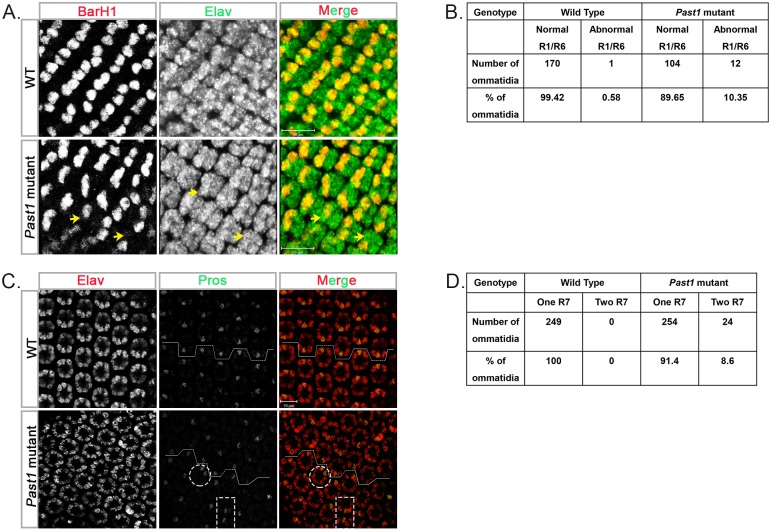
Specification of R1/R6 and R7 photoreceptors in *Past1* mutant eyes. (A) BarH1 (grey, or red in the merge) and Elav (grey, or green in the merge) staining of wild type and *Past1*^*110-1*^ homozygous mutant eye discs from third instar larvae. Shown are Z-projections of confocal sections. The yellow arrow marks the missing photoreceptors. (B) Elav (grey, or red in the merge) and pros (grey, or green in the merge) staining of wild type and *Past1*^*110-1*^mutant early-mid pupa eyes (42-48h after puparium formation). The dotted line marks the equator of the eye, defining the dorsal and ventral parts of the eye. The dotted circle delineates an ommatidium with two R7 photoreceptors and the dotted rectangle outlines two neighboring ommatidia that face each other in the same dorsal part of the eye. (C) Quantification of ommatidia containing one or two R7 photoreceptor in wild type (n = 249) and *Past1*^*110-1*^mutant (n = 278) ommatidia; *P* < 0.001.

The first photoreceptor to differentiate in the developing fly eye is R8 [18, 34]. To determine whether PAST1 plays a role in R8 differentiation, eye discs of third instar *Past1* mutant larvae were immunostained with an antibody against the R8-specific transcription factor senseless (sense) [34, 35]. No abnormalities in the number of R8 photoreceptors were detected (Fig 3A), implying that differentiation of R8 is unaffected by the absence of PAST1. Since there were no indications for abnormality in differentiation of R2/R5 and of R3/R4 photoreceptors, we tested whether there are any defects in the next stage of differentiation, namely, development of R1/R6 photoreceptors. We first stained late pupal eyes with anti-Rhodopsin 1 (Rh1) antibody, which recognizes rhabdomeres R1-R6 only [14]. The results revealed an occasional absence of either one or two photoreceptors. According to their localization within the developing ommatidia, they could have been either R1 or R6 photoreceptors, or both (Fig 3B). More so, in some of these ommatidia, there was a duplication of what looked like R7 photoreceptor (Fig 3B-V). To directly test whether the absence of *Past1* leads to improper development of photoreceptors R1, R6 and R7, eye discs of third instar larvae of *Past1* mutant were stained with anti-BarH1 antibodies. BarH1 is expressed in the nuclei of photoreceptors R1 and R6, especially during the third instar larval stage (Higashijima et al 1992; Charlton-Perkins et al., 2011). The results, presented in Fig 4A and 4B, indicated that in 10% of the ommatidia derived from *Past1* mutant eye discs there was only one R1/R6 instead of two photoreceptors.

In light of the aberrant differentiation of R1/R6 photoreceptors, we wondered whether lack of PAST1 affects the differentiation of R7. To that end, early pupal eyes were immunostained with anti-prospero (pros), which specifically marks R7 nuclei as well as cone cells. To confirm visualization of the R7 confocal plane and not that of cone cells, we co-stained the samples with anti-Elav antibody such that co-localization of pros and Elav represents an R7 cell. The results (Fig 4C and 4D) showed that 8.6% of the ommatidia in early pupal *Past1* mutant eyes contained two R7 photoreceptors instead of the single R7 photoreceptor normally present in each ommatidium.

**Fig 5:**
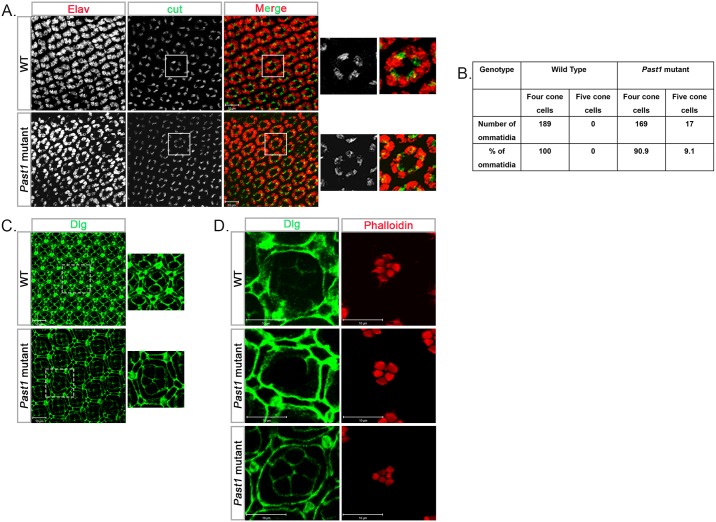
Specification of cone and inter-ommatidial cells in *Past1* mutant eyes. (A) Elav (grey, or red in the merge) and cut (grey, or green in the merge) staining of wild type and *Past1*^*110-1*^ homozygous mutant early-mid pupal eyes (42-48h after puparium formation). Shown are Z-projections of confocal sections. The enlarged images on the right of each frame show an ommatidium with four cone cells in wild type and an ommatidium with five cone cells in *Past1*^*110-1*^mutant eyes. (B) Quantification of four or five cone cells in wild type (n = 189) and *Past1*^*110-1*^mutant (n = 186) ommatidia; *P* < 0.001. (C) Dlg (green) staining of wild type and *Past1*^*110-1*^mutant early-mid pupal eyes (42-48h after puparium formation). Shown are Z-projections of confocal sections. The enlarged images on the right of each frame show an ommatidium with nine inter-ommatidial pigment cells and four cone cells in wild type and an ommatidium with nine inter-ommatidial pigment cells and five cone cells in *Past1*^*110-1*^mutant eyes. (D) Dlg (green) and phalloidin (red) staining of wild type and *Past1*^*110-1*^mutant late pupal eyes (85-96h after puparium formation). Different focal planes of the same ommatidium, presenting: upper sections of the ommatidia for Dlg and lower sections for phalloidin.

We also followed the development of the four non-neuronal cone cells, which are specified after R7 differentiation in early-mid pupae. Staining with anti-cut antibody revealed that 9.1% of *Past1* mutant ommatidia had five cone cells instead of the normal four (Fig 5A and 5B). Interestingly, the improper development of the photoreceptors and cone cells in the *Past1* mutant was not associated with an abnormal development of the IPC, as detected by immunostaining of early-mid and late pupa stages with anti-Discs large (Dlg) antibodies, which mark the cellular membrane of these cells (Fig 5C and 5D).

Taken together, our results show that PAST1 is important for normal development of photoreceptors R1/R6/R7, as well as of cone cells.

**Fig 6:**
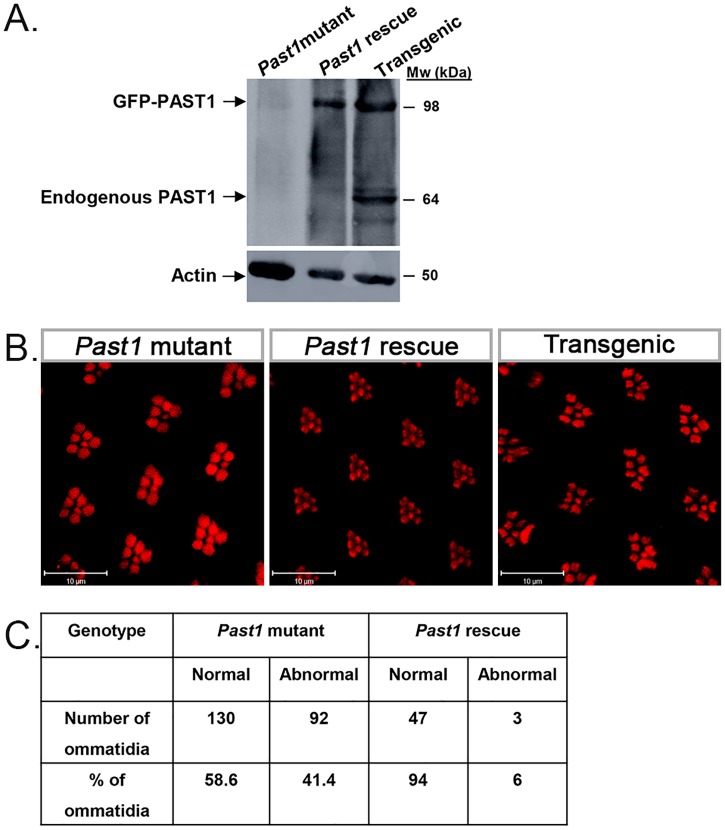
Rescue of ommatidial defects in *Past1* mutant by PAST1B transgenic expression. (A) Lysates of one day old adult fly heads with the following genotypes: 1) GMRGal4>Sco/CyO; *Past1*^*110-1*^/*Past1*^*110-1*^ (negative control), 2) GMRGal4>GFP-PAST1B/CyO; *Past1*^*110-1*^/*Past1*^*110-1*^ (rescue), 3) GMRGal4>GFP-PAST1B/CyO; Sb/TM6b (transgenic/GFP-PAST1), were subjected to SDS-PAGE. The corresponding blot was interacted with anti-PAST1 serum and anti-actin antibody. (B) Phalloidin (red) staining of homozygous *Past1*^*110-1*^, GMRGal4>GFP-PAST1B/CyO; *Past1*^*110-1*^/ *Past1*^*110-1*^ and GMRGal4>GFP-PAST1B/CyO; Sb/TM6b, late pupa eyes (85-96h after puparium formation). (C) Quantification of rhabdomeres number in late pupa eyes of *Past1*^*110-1*^ homozygous mutant (n = 222) and GMRGal4>GFP-PAST1B/CyO; *Past1*^*110-1*^/ *Past1*^*110-1*^ (rescue) (n = 50) ommatidia; *P* < 0.001flies.

To demonstrate that the described *Past1* null phenotype was indeed a result of PAST1 loss, a rescue experiment was performed using PAST1B transgenes (Fig 6A–6C). Homozygous null *Past1* mutant flies expressing a GFP-PAST1B transgene at comparable levels in their eyes (see Fig 6A) displayed only 6% of abnormal ommatidia with less than seven rhabdomeres (Fig 6B and 6C). This result strongly implies that the absence of PAST1 in the *Past1* null mutant flies leads to abrogated development of their eyes.

### Transgenic PAST1 has a dominant negative effect on eye development

**Fig 7:**
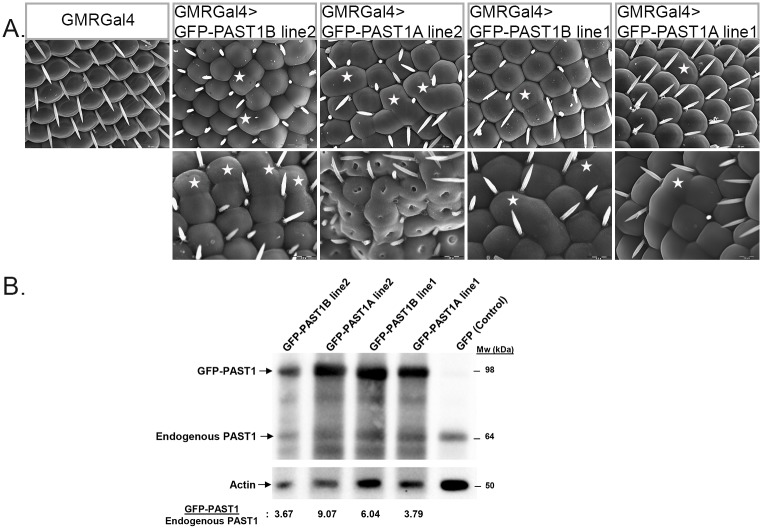
Overexpression of PAST1 in the eyes results in fused ommatidia. (A) Scanning electron microscopy images show eyes of three-days-old adult flies. Upper panels: GMRGal4 (control), GMRGal4>UAS-GFP-PAST1B/TM6B (line 2), GMRGal4>UAS-GFP-PAST1A/TM6B (line 2), GMRGal4>UAS-GFP-PAST1B/CyO (line 1) and GMRGal4>UAS-GFP-PAST1A/CyO (line 1). Lower panels: GMRGal4>UAS-GFP-PAST1B/UAS-GFP-PAST1B (line 2), GMRGal4>UAS-GFP-PAST1A/UAS-GFP-PAST1A (line 2), GMRGal4>UAS-GFP-PAST1B/UAS-GFP-PAST1B (line 1) and GMRGal4>UAS-GFP-PAST1A/UAS-GFP-PAST1A (line 1). Differences in bristle numbers were not taken into account since they also appear in the UAS-GFP control flies. (B) Lysates from GMRGal4>UAS-GFP-PAST1B/UAS-GFP-PAST1B (line 2), GMRGal4>UAS-GFP-PAST1A/UAS-GFP-PAST1A (line 2), GMRGal4>UAS-GFP-PAST1B/UAS-GFP-PAST1B (line 1), GMRGal4>UAS-GFP-PAST1A/UAS-GFP-PAST1A (line 1) and GMRGal4>UAS-GFP (control) adult flies were subjected to SDS-PAGE and western blotting using anti-PAST1 and anti-actin antibodies. Quantification of GFP-PAST1 expression is shown at the bottom of the western blotting. In each lane, the band intensity of GFP-PAST1 expression was divided by the intensity of endogenous PAST1 band, both detected using anti-PAST1 antibodies. The results are the mean of four different experiments. Stars denote fusion between ommatidia.

To further explore the effect of PAST1 on eye development we tested transgenic flies overexpressing GFP-PAST1A or GFP-PAST1B in their eyes under the control of GMR-Gal4 (see Fig 7). While *Past1* mutant flies did not display any external abnormalities in the morphology of the adult eye (Fig 1C), the eyes of flies overexpressing either PAST1A or PAST1B exhibited ommatidial fusion in various degrees of severity (Fig 7A). The severity of eye defects was in correlation with the level of transgenic PAST1 expression (Fig 7B).

**Fig 8:**
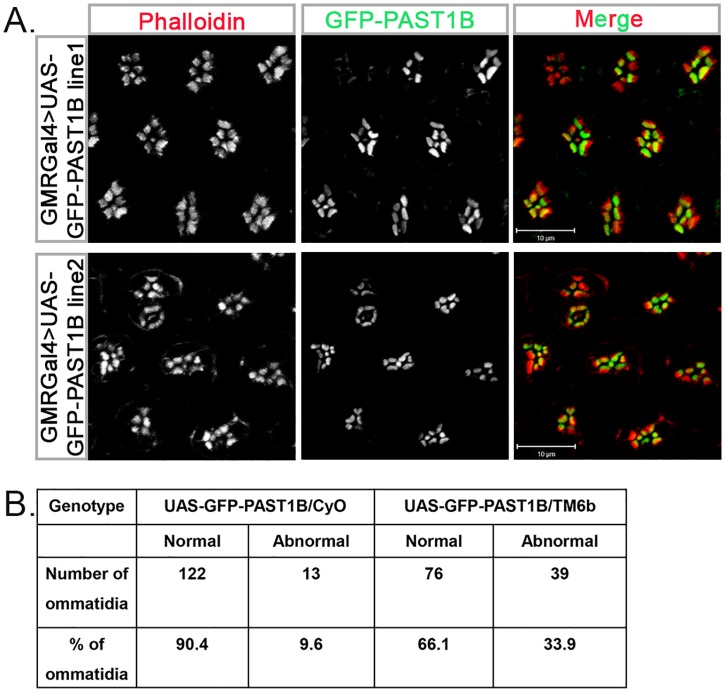
Transgenic GFP-PAST1B contains an abnormal number of photoreceptors. (A) Phalloidin (grey, or red in the merge) staining of GMRGal4>UAS-GFP-PAST1B/CyO (line 1) or GMRGal4>UAS-GFP-PAST1B/TM6b (GFP appears as grey, or green in the merge) (line 2) late pupal eyes (85-96h after puparium formation). (B) Quantification of abnormal ommatidia (ommatidium with more or less seven rhabdomeres) in late pupal eyes of PAST1B (line 1) (n = 135) or PAST1B (line 2) (n = 115) ommatidia; *P* < 0.001 only for PAST1B (line 2).

Since endogenous PAST1A is not expressed in the fly eye [2] we focused our further analysis on PAST1B. To analyze the development of photoreceptors in the presence of transgenic PAST1B, two lines were chosen, one with a high expression level of the transgene (line 2) and the other (line 1) presents a low expression level (Fig 7). Phalloidin-stained late pupal GFP-PAST1B eyes displayed abnormal number of rhabdomeres (Fig 8A and 8B). Overall, 10% of the ommatidia in the GFP-PAST1B line 1 and 34% of the ommatidia in the GFP-PAST1B line 2 flies were abnormal (Fig 8B).

**Fig 9:**
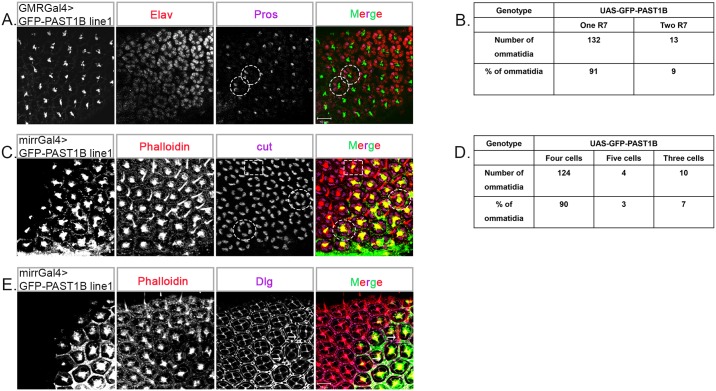
Specification of R7, cone and inter-ommatidial pigment cells in GFP-PAST1B transgenic flies. (A) Staining of GMRGal4>UAS-GFP-PAST1B (GFP appears as grey, or green in the merge, in all the panels) (line 1), early-mid pupa eyes (42-48h after puparium formation) with Elav (grey, or red in the merge) and pros (grey, or purple in the merge). The dotted circle represents an ommatidium with two R7 photoreceptors (B) Quantification of the number of R7 photoreceptors in early-mid pupa eyes of wild type (n = 249) and overexpressing transgenic PAST1B (line 1) (n = 145) ommatidium; *P* < 0.001. (C) Phalloidin (grey, or red in the merge) and cut (grey or purple in the merge) staining of mirrGal4>UAS-GFP-PAST1B (line 1) early-mid pupa eyes (42-48h after puparium formation). The dotted circles represent ommatidia with five cone cells and the dotted square represents an ommatidium with three cone cells. Shown are Z-projections of confocal sections. (D) Quantification of the number of cone cells in early-mid pupal eyes of wild type (n = 189) and transgenic PAST1B (line 1) (n = 138) ommatidia; *P* < 0.002. (E) Phalloidin (grey, or red in the merge) and Dlg (grey, or purple in the merge) staining of mirrGal4>UAS-GFP-PAST1B (line 1) early-mid pupa eyes (42-48h after puparium formation). The arrowhead marks an extra inter-ommatidial pigment cell.

Staining with anti-pros antibody in early pupal eyes of GFP-PAST1B line 1 under the control of GMR-Gal4 resulted in 9% of the ommatidia containing two R7 photoreceptors (Fig 9A and 9B). We employed an additional driver, mirr-Gal4, whose expression is limited to the dorsal area of the eye, thus enabling comparison between the dorsal region overexpressing GFP-PAST1B and the ventral region, used as a control. Transgenic expression of GFP-PAST1B line 1 also led to abnormal differentiation of the cone cells. Instead of four cone cells, we observed either three or five cone cells in 10% of the GFP-PAST1B overexpressing ommatidia (Fig 9C and 9D). Abnormalities were observed in the inter-pigment cells as well, in the form of an extra IPC (Fig 9E), a phenomenon not observed in the eyes of the *Past1* null mutant flies (Fig 5C and 5D).

In summary, our results indicated that overexpression of PAST1B in the fly eye leads to defects in the development of photoreceptors, cone cells and IPC cells.

### PAST1 modulates endocytosis

**Fig 10:**
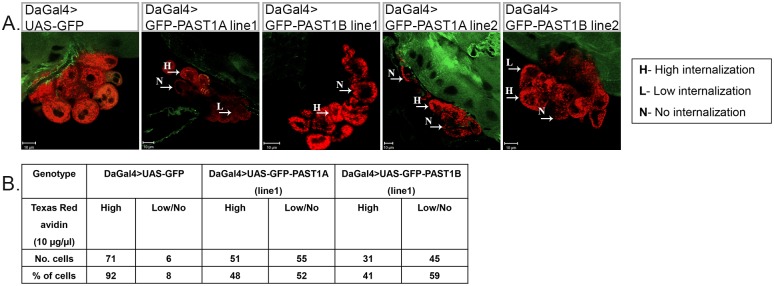
Overexpression of PAST1 attenuates endocytosis in Garland cells. (A) Garland cells from third instar larvae of the following genotypes were dissected and incubated with Texas-red-conjugated avidin for one minute following 20 minutes of chase, fixed and visualized: DaGal4>UAS-GFP (control), DaGal4>UAS-GFP-PAST1A (line 1), DaGal4>UAS-GFP-PAST1B (line 1), DaGal4>UAS-GFP-PAST1A (line 2) and DaGal4>UAS-GFP-PAST1B (line 2). Cells were classified into: H—high level of internalization of avidin (wild type level of endocytosis), L—low level of internalization of avidin (reduced or no endocytosis), N—no internalization of avidin. (B) Quantification of avidin intensity in Garland cells of DaGal4>UAS-GFP (control) (n = 77), DaGal4>UAS-GFP-PAST1A (line 1) (n = 106), *P* < 0.002 and DaGal4>UAS-GFP-PAST1B (line 1) (n = 76); *P* < 0.001.

We have previously shown that the absence of PAST1 leads to decreased endocytosis of fluorescently labeled Texas-Red avidin in Garland cells [2]. In light of this finding, we aimed to assess the effect of PAST1 overexpression on endocytosis using the same assay. As presented in Fig 10A, avidin was readily endocytosed by all wild-type Garland cells. However, Garland cells isolated from GFP-PAST1A (line 1) or GFP-PAST1B (line 1) overexpressing third instar larvae displayed attenuated endocytosis (Fig 10A). The Garland cells in these larvae could be classified into three groups: cells that endocytosed avidin similarly to wild-type cells, cells that presented reduced level of avidin endocytosis and cells that did not display any endocytosis of avidin (Fig 10A). As summarized in Fig 10B, overexpressed PAST1 had a dominant negative effect with approximately 50%-60% attenuation of endocytosis. To summarize, our results strongly indicated that overexpression of PAST1 attenuated endocytosis of fluorescently labeled avidin.

Since Boss, expressed on the surface of R8 photoreceptor, is internalized by the neighboring R7 cell [20–24], we tested whether this endocytic process is also impaired in eyes of *Past1* mutant and transgenic flies. To do so, we immunostained eye discs from *Past1* null and PAST1 overexpressing flies with anti-Boss antibody. The results, presented in S1 Fig, indicated no change in Boss staining of R7 cells of mutant or transgenic GFP-PAST1B eye discs.

## Discussion

In the present work we utilized the *Drosophila* eye to study the physiological role of the endocytic protein PAST1 during development. Recently it has been shown that EHD1 is necessary for proper differentiation of ocular tissues including the lens, cornea and retina in mice [29].

In the *Past1* null mutant flies no external eye abnormalities were observed, but the development of photoreceptors R1, R6 and R7, as well as of cone cells, was abnormal. In flies overexpressing PAST1 in the eye external abnormalities were noted, as well as an abnormal number of photoreceptors, cone cells and IPCs. Defects in ommatidium development accompanied by no external abnormalities in the eye have been previously reported for other mutations. For example, the external eye of mutants in *Muscleblind* (*Mbl*), a regulator of alternative splicing, appear to be normal, yet tangential sections revealed that they harbored ommatidia defects [36]. Another example involves *Crumbs* (*Crb*), a transmembrane protein which is essential for biogenesis of adherens junctions and for establishing apical-basal polarity in *Drosophila* epithelia by downregulating endocytosis of Notch and Delta during eye development [4, 37–39]. External morphology of mutant *Crb* (*crb*^*S87-2*^) was reported to be normal. However, internal structure of adult *Crb*^*S87-2*^ mutant ommatidia was found to be defective with shortened and bulkier rhabdomeres, often in contact with each other rather than being distinct as in wild type eyes [38].

Interestingly in *Past1* null flies, the ommatidia always contained at least one R7 photoreceptor, with some having two R7 photoreceptors with a concomitant loss of either R1 or R6 or both. These results imply that differentiation to R1 or R6 was abrogated and instead, at least one R7 photoreceptor was developed. No change in Boss staining was detected in R7 cells of eye discs from *Past1* null mutants or from transgenic GFP-PAST1 flies (S1 Fig), while in *Past1* null mutants the ommatidia always contained at least four cone cells and some ommatidia had five cone cells.

We have previously shown that *Past1* genetically interacts with Notch in the wing [2]. The results of the present study indicated that *Past1* plays a role during eye development, which is known to be regulated by Notch [17, 19, 27, 40]. Interestingly, endocytosis of Notch seemed abnormal in early-mid pupal mutant eyes. Thus, higher level of Notch staining was detected at the vicinity of the plasma membrane of mutant pigment cells, indicating abrogated endocytosis (S2 Fig). This result implies abnormal internalization of Notch in the absence of PAST1 and hints to the possibility that PAST1 modulates internalization of Notch during eye development.

**Fig 11:**
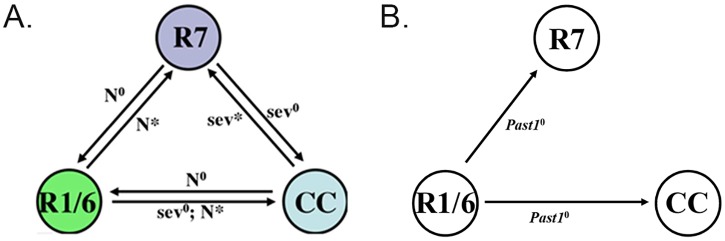
A model for ommatidium development. (A) According to Tomlinson et al. 2011, expression of Notch in R1 or R6 leads to their development into R7 or cone cells. (B) A model for ommatidium development, presenting the impact of *Past1* mutation (*Past1*^*null*^) on development of R1/R6/R7 and the cone cells. We assume that when overexpressed there is PAST1-mediated downregulation of endocytic processes, as shown in Fig 10, and therefore it behaves like a mutant *Past1*, that leads to Notch overexpression.

Our results well fit the model suggested by Tomlinson et al., (2011) for the development of photoreceptors R1/R6/R7 and cone cells. According to this model Notch overexpression in either R1 or R6 photoreceptors, leads to development of one of these cells into an extra R7 photoreceptor. Additionally, overexpression of Notch in R1 or R6 combined with an absence of Sev leads to the appearance of an extra cone cell [17]. We propose that lack of PAST1 elevates Notch activation in R1 and R6 photoreceptors, which subsequently develop into R7 or into cone cells (Fig 11). We hypothesize that PAST1 negatively regulates Notch signaling in R1 and R6. Since penetrance of *Past1* mutation in the eye is low, PAST1 is, most probably, not a major regulator of Notch signaling, but fine-tunes it. Further experiments are needed to establish the role of PAST1 in endocytosis of Notch.

Eyes of flies overexpressing PAST1 were rough with regions of fused ommatidia (Fig 7A) and exhibited extra IPCs (Fig 9E). Mutations that abrogate the normal cell death process of IPCs have been shown to lead to rough eyes [25, 41]. More so, overexpression of Notch (N^FL^ or N^ICD^) in the pupal retina led to appearance of extra IPCs [27, 42–44]. Therefore, we assume that the rough eyes phenotype in the PAST1 transgenic flies is due to abnormal PCD of IPCs.

In the present study we examined the ability of Garland cells overexpressing PAST1 to internalize fluorescently labeled Texas-Red avidin, as was examined for *Past1* mutants [2]. Garland cells overexpressing *Past1* displayed attenuated endocytosis. Overexpression of PAST1 had a dominant negative effect, showing approximately 50–60% attenuation in endocytosis (Fig 10). A similar dominant negative effect has also been previously shown in HeLa cells overexpressing the mammalian EHD2, leading to attenuation of plasma membrane internalization [45].

In conclusion, the results presented in this work highlight the importance of PAST1 for development of R1,R6 and R7 photoreceptors of the fly ommatidia.

## Supporting Information

S1 Fig Normal internalization of Boss into R7 photoreceptor in *Past1* mutant and *Past1* transgenic eye disc. Boss (grey) staining of wild type, *Past1*^*110-1*^ mutant and GMRGal4>UAS-GFP-*Past1*B larval eye discs. Shown are Z-projections of confocal sections.
(DOCX)

S2 Fig Notch upregulated in *Past1* mutant pupal eye. (A) Notch intracellular domain (green) staining of wild type and *Past1*^*110-1*^ homozygous mutant early-mid pupal eyes (42-48h after puparium formation). (B) Quantification of fluorescent intensity in the pupal eyes of wild type and *Past1*^*110-1*^ homozygous mutant. Results represent the mean ± SD of 26 eyes of wild type and *Past1*^*110-1*^ homozygous mutant from five independent experiments, statistically analyzed using the student *t*-test.
(DOCX)
