# Correction: *Past1* Modulates *Drosophila* Eye Development

**DOI:** 10.1371/journal.pone.0174495

**Published:** 2017-03-20

**Authors:** Orly Dorot, Hermann Steller, Daniel Segal, Mia Horowitz

S1 Fig and S2 Fig are incorrect. Please view the correct S1 Fig and S2 Fig below.

## Supporting information

S1 Fig Normal internalization of Boss into R7 photoreceptor in *Past1* mutant and *Past1* transgenic eye disc. Boss (grey) staining of wild type, *Past1*^*110-1*^ mutant and GMRGal4>UAS-GFP-*Past1*B larval eye discs. Shown are Z-projections of confocal sections.
(TIF)

S2 Fig Notch upregulated in *Past1* mutant pupal eye. (A) Notch intracellular domain (green) staining of wild type and *Past1*^*110-1*^ homozygous mutant early-mid pupal eyes (42-48h after puparium formation). (B) Quantification of fluorescent intensity in the pupal eyes of wild type and *Past1*^*110-1*^ homozygous mutant. Results represent the mean ± SD of 26 eyes of wild type and *Past1*^*110-1*^ homozygous mutant from five independent experiments, statistically analyzed using the student *t*-test.
(TIF)
