# Supplementary material for: Past1 Modulates Drosophila Eye Development
Source: PLoS One. 2017 Jan 6;12(1):e0169639. doi: 10.1371/journal.pone.0169639 (PMC5218476; doi:10.1371/journal.pone.0169639)
Supplement: S1 Fig — Boss (grey) staining of wild type, Past1110-1 mutant and GMRGal4>UAS-GFP-Past1B larval eye discs. Shown are Z-projections of confocal sections. (DOCX) [file pone.0169639.s001.docx]

**S1 Fig. Normal internalization of Boss into R7 photoreceptor in *Past1* mutant and *Past1* transgenic eye disc.**

Boss (grey) staining of wild type, *Past1^110-1^* mutant and GMRGal4>UAS-GFP-*Past1*B larval eye discs. Shown are Z-projections of confocal sections.
