# Supplementary material for: Past1 Modulates Drosophila Eye Development
Source: PLoS One. 2017 Jan 6;12(1):e0169639. doi: 10.1371/journal.pone.0169639 (PMC5218476; doi:10.1371/journal.pone.0169639)
Supplement: S2 Fig — (A) Notch intracellular domain (green) staining of wild type and Past1110-1 homozygous mutant early-mid pupal eyes (42-48h after puparium formation). (B) Quantification of fluorescent intensity in the pupal eyes of wild type and Past1110-1 homozygous mutant. Results represent the mean ± SD of 26 eyes of wild type and Past1110-1 homozygous mutant from five independent experiments, statistically analyzed using the student t-test. (DOCX) [file pone.0169639.s002.docx]

**S2 Fig.** **Notch upregulated in *Past1* mutant pupal eye.**

(A) Notch intracellular domain (green) staining of wild type and *Past1^110-1^* homozygous mutant early-mid pupal eyes (42-48h after puparium formation). (B) Quantification of fluorescent intensity in the pupal eyes of wild type and *Past1^110-1^* homozygous mutant. Results represent the mean ± SD of 26 eyes of wild type and *Past1^110-1^* homozygous mutant from five independent experiments, statistically analyzed using the student *t*-test.
