# Supplementary figures and images for: Correction: Past1 Modulates Drosophila Eye Development
Source: PLoS One. 2017 Mar 20;12(3):e0174495. doi: 10.1371/journal.pone.0174495 (PMC5358843; doi:10.1371/journal.pone.0174495)

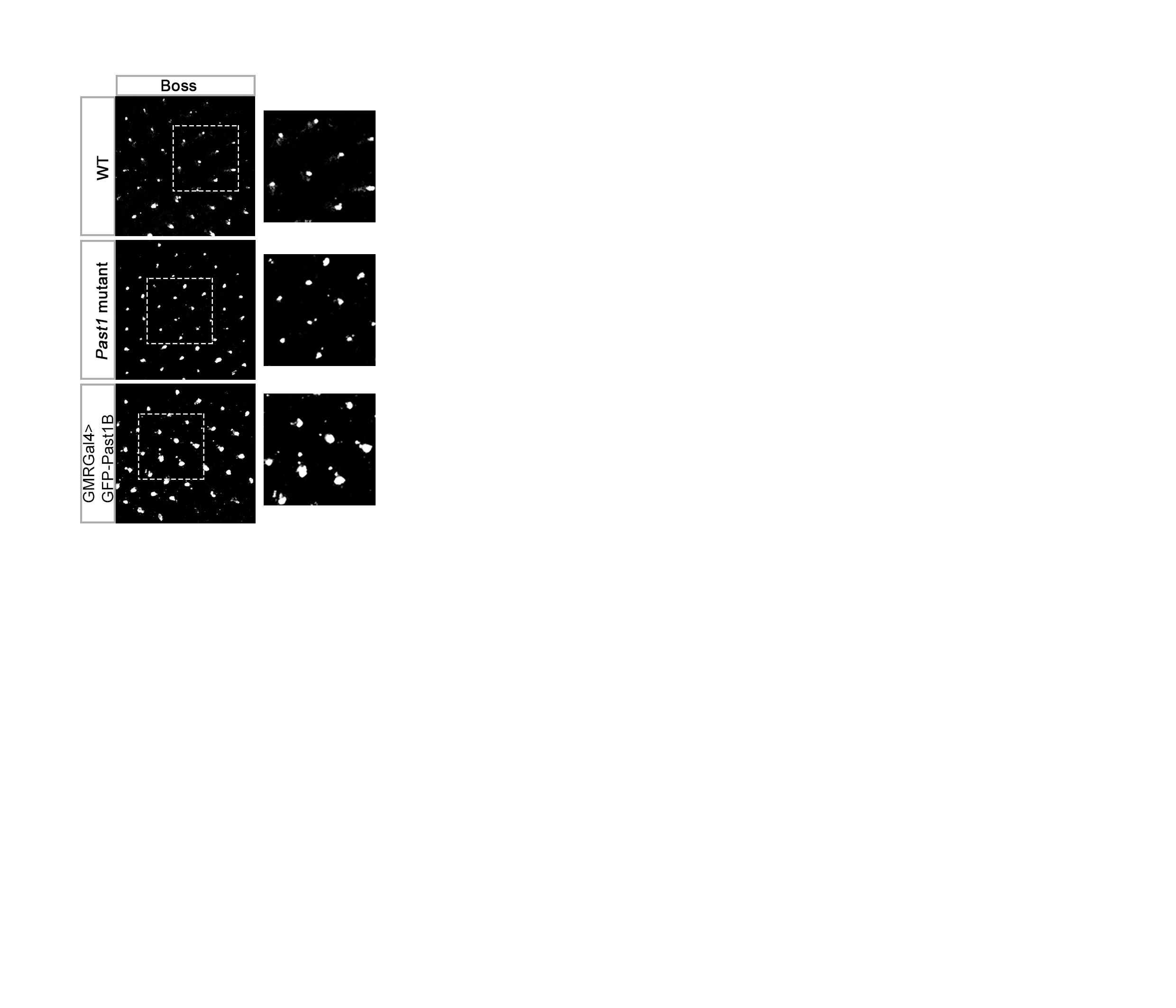

Supplement: S1 Fig — Boss (grey) staining of wild type, Past1110-1 mutant and GMRGal4>UAS-GFP-Past1B larval eye discs. Shown are Z-projections of confocal sections. (TIF) [file pone.0174495.s001.tif]

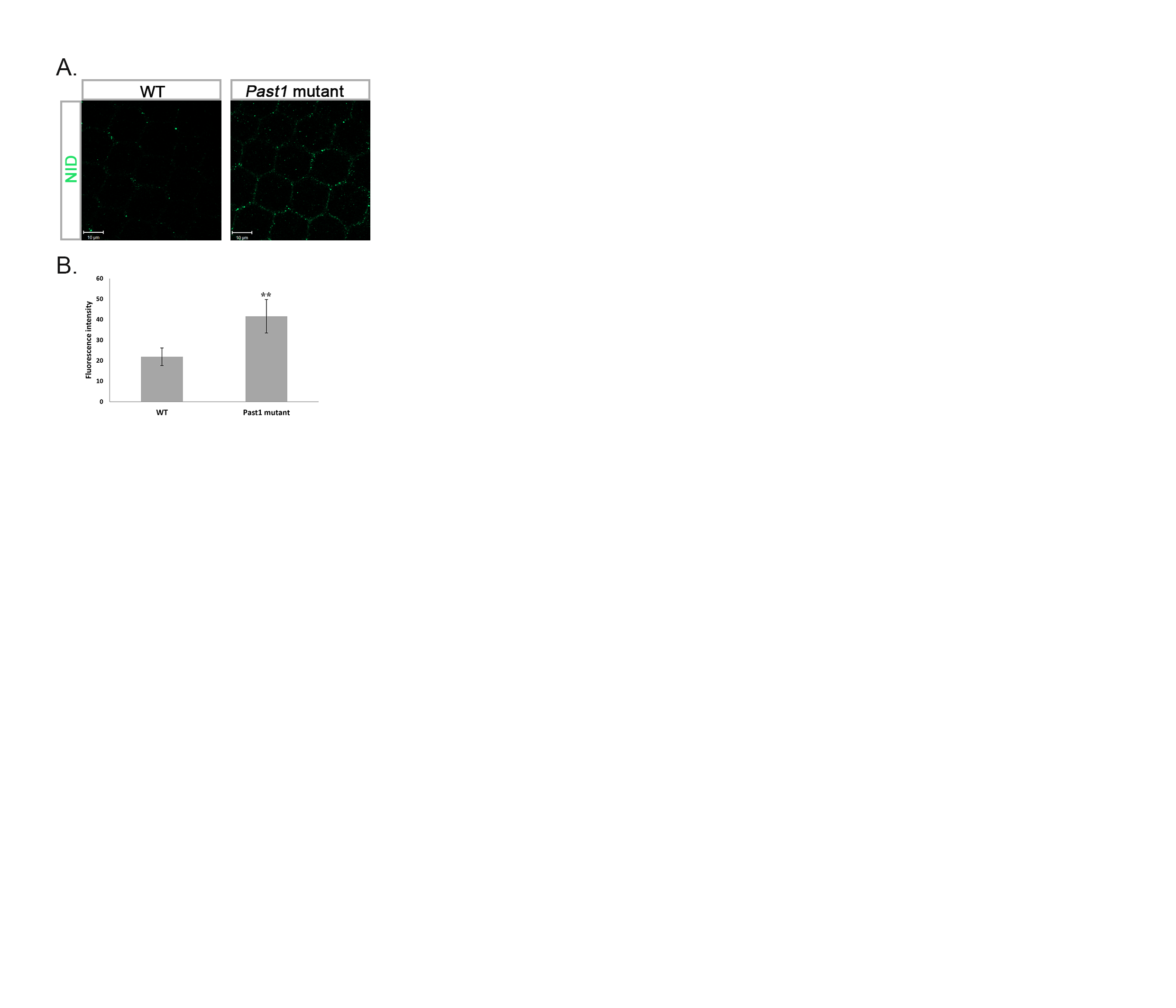

Supplement: S2 Fig — (A) Notch intracellular domain (green) staining of wild type and Past1110-1 homozygous mutant early-mid pupal eyes (42-48h after puparium formation). (B) Quantification of fluorescent intensity in the pupal eyes of wild type and Past1110-1 homozygous mutant. Results represent the mean ± SD of 26 eyes of wild type and Past1110-1 homozygous mutant from five independent experiments, statistically analyzed using the student t-test. (TIF) [file pone.0174495.s002.tif]
